# Bioactive Carbohydrates and Peptides in Foods: An Overview of Sources, Downstream Processing Steps and Associated Bioactivities

**DOI:** 10.3390/ijms160922485

**Published:** 2015-09-17

**Authors:** Maria Hayes, Brijesh K. Tiwari

**Affiliations:** The Food BioSciences Department, Teagasc Food Research Centre, Ashtown, Dublin 15, Ireland; E-Mail: brijesh.tiwari@teagasc.ie

**Keywords:** peptides, angiotensin-I-Converting enzyme (ACE-I), renin, platelet activating factor acetylhydrolase (PAF-AH), downstream processing, carbohydrates, chitin, fucan, algaran, ulvans, chitosan, mental health, diabetes, prebiotics

## Abstract

Bioactive peptides and carbohydrates are sourced from a myriad of plant, animal and insects and have huge potential for use as food ingredients and pharmaceuticals. However, downstream processing bottlenecks hinder the potential use of these natural bioactive compounds and add cost to production processes. This review discusses the health benefits and bioactivities associated with peptides and carbohydrates of natural origin and downstream processing methodologies and novel processes which may be used to overcome these.

## 1. Introduction

Carbohydrates play an essential role in human biology and disease development and are a relatively untapped source of bioactive compounds for use as functional foods or pharmaceuticals. In contrast, bioactive peptides or “cryptides” have experienced an explosion of scientific research in recent decades and an impressive array of health attributes have been assigned to peptides generated from food protein sources including dairy, marine, plants and seeds. Bioactive peptides or “cryptides” are sequences of approximately 2–20 amino acids in length that impart a positive health effect to the consumer which goes above and beyond basic human nutrition [1]. They must be bioavailable and capable of exerting this health effect at their target site in the gut, bloodstream or elsewhere [2]. A myriad of positive health beneficial properties are associated with bioactive peptides including antihypertensive, anti-diabetic, anti-obesity, immune-modulatory, relaxing and satiety inducing effects [3]. Furthermore, bioactive peptides can also be generated from meat and underutilized by-products or processing waste/discards produced as a result of food processing [4]. Indeed, bioactive peptides can result from food processing steps including fermentation, high temperature treatment, and pasteurization and cooking [4]. Bioactive peptides derived from natural sources generally act at higher concentrations than their synthetic drug counterparts and functional foods should thus be used for disease prevention rather than treatment. Despite this, bioactive peptides used as functional food ingredients do not accumulate in body tissue [5] and there are only a few reports regarding negative side effects when bioactive peptides are used for preventative healthcare purposes [3]. Bottlenecks in the development of bioactive peptides and their use in food and cosmetic products include costs associated with downstream processing steps, bioavailability of the bioactive peptide and compliance with European Food Safety Authority (EFSA) health claim regulations and other regulatory bodies including the Food Development Authority (FDA) and the Foods of Specific Health Use (FOSHU) system in Japan [6]. Moreover, substantiation of health claims associated with bioactive peptides derived from food sources in the past did not provide enough clinical evidence of the claimed health effect and often the mechanisms of action were not determined [7].

Research efforts concerning the use of bioactive carbohydrates or polysaccharide in functional foods as well as oligosaccharides are still considered under-exploited. However, polysaccharides from natural sources including those isolated from marine and dairy sources have found use in a number of biotechnological and pharmaceutical applications. For example, the polysaccharide chitin which may be generated from prawn and crab shell material and Basidomycete mushrooms, and its de-acetylated form chitosan, have found application as polymers for use in encapsulation technologies [8]. Chitin and chitosan have also been examined for their use as functional food ingredients and have demonstrated anti-obesity and satiety effects in previous studies [8,9]. Furthermore, chitosan is a known coagulant and is used in the manufacture of medical bandages [8,9]. More recently, the prebiotic effects of chitin and chitosan as well as other polysaccharides derived from brown, red and green macroalgae including fucoidan, alginate and ulvans were examined [5,10]. Polysaccharides and oligosaccharides from dairy sources such as yoghurt usually originate from the Generally Recognized as Safe (GRAS) exo-polysaccharide producing bacteria present in the product [11]. Since the dietary fiber intake of many people is below their suggested adequate intake values, strategies to successfully fortify foods with fiber is an important area of food research. In order to provide a source of fiber to the consumer, plant derived polysaccharides may be added to dairy products and sources can include soy and cereals [12]. This paper collates information concerning the varied sources of bioactive carbohydrates and peptides, methods used for purification and downstream processing steps, the bioactivities of these bioactive compounds, their mechanism of action, and bottlenecks concerning their future development.

## 2. Bioactivities Associated with Peptides and Carbohydrates

Food derived bioactive peptides refer to compounds from animal and plant sources generated by food processing, fermentation, enzymatic or chemical hydrolysis or gastrointestinal digestion and which have regulatory functions in the human system beyond normal and adequate nutrition [13]. Table 1 lists bioactive peptides discovered from a number of sources including soy, wheat, dairy, marine resources including fish processing co-products, meat and others and the commercial products in which they are found. A number of activities have been described for bioactive peptides including antimicrobial, blood-pressure lowering including angiotensin converting enzyme-1 (ACE-I) and renin inhibitory bioactivities, anti-atherosclerotic, antioxidant, antithrombotic, enhancement of mineral absorption, immune-modulatory and opioid activities. Often peptides can have several bioactivities, are multifunctional and exert more than one effect [14].

### 2.1. Heart Health and Coagulation Beneficial Peptides

High blood pressure or hypertension is the major risk factor for myocardial infarction, congestive heart failure, arteriosclerosis, and stroke and end-stage renal disease. The enzymes angiotensin converting enzyme I (ACE-I; EC 3.4.15.1) and renin (EC 3.4.23.15) play an important role in the control and regulation of blood pressure and salt water balance within the renin angiotensin aldosterone system (RAAS) [15]. ACE-I is the main target in treatment of high blood pressure and several synthetic drugs including captopril (Capoten^®^), lisinopril and enalapril are currently used as pharmaceuticals to treat this problem [15]. However, these drugs have adverse side effects including sleep apnea, dry cough, angioedema and others [16,17]. Food derived bioactive peptides have shown potential for use as mild or moderate ACE-I and renin inhibitory peptides and several of these are documented in the database BIOPEP [15].

#### 2.1.1. Sources and Structure of ACE-I Inhibitory Peptides

**Figure 1:**
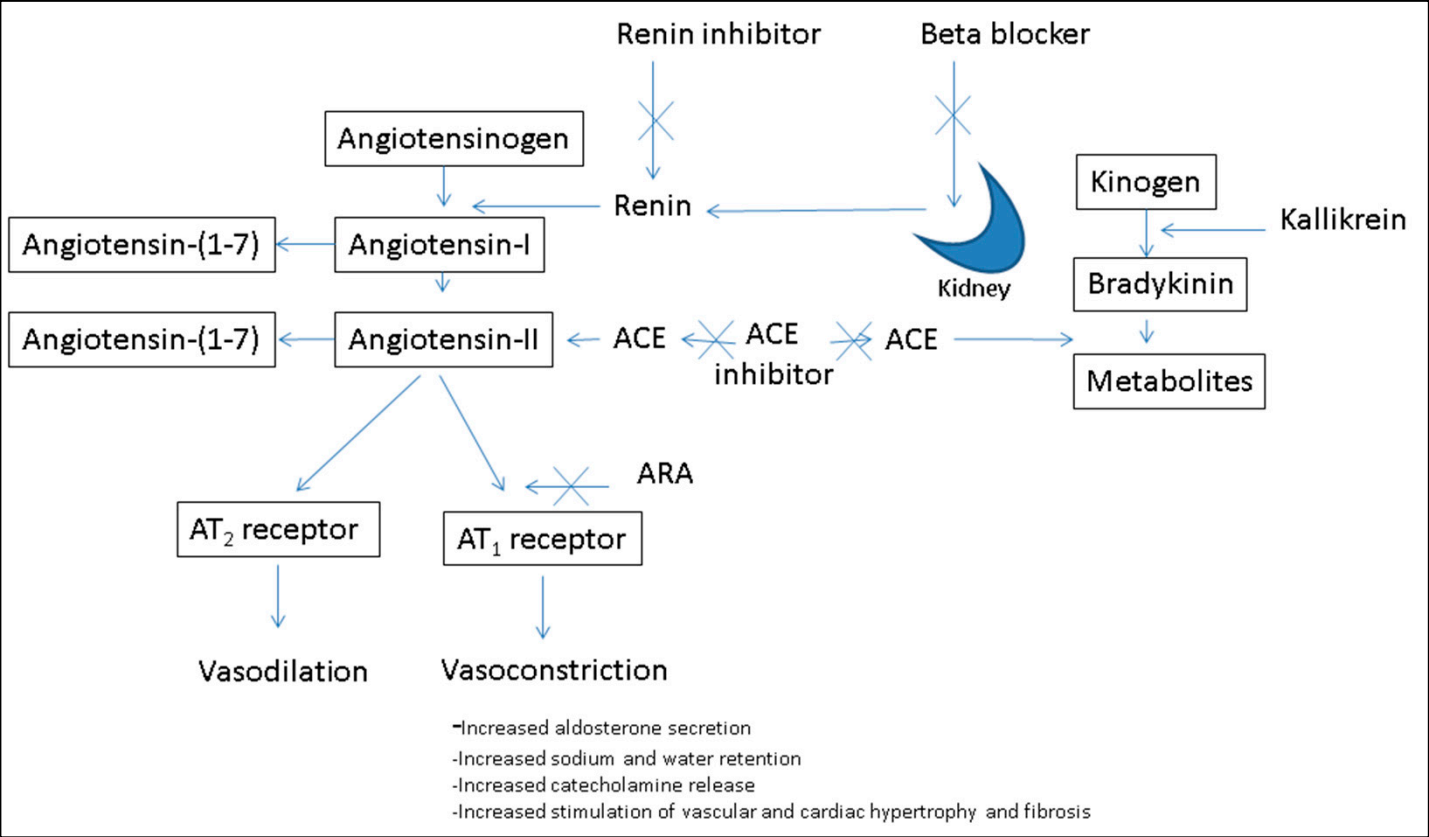
The Renin-Angiotensin-Aldosterone System (RAAS) can be inhibited by ACE-I inhibitors, angiotensin II type 1 receptor antagonists (ARA), renin inhibitors and beta blockers. ACE-I also plays a role in bradykinin metabolism and metabolism of angiotensin-(1–7).

ACE-I inhibitory peptides were first identified by the British scientist Sir John Vane who observed the vasodilatory effect of snake venom [18]. ACE-I catalyzes the conversion of the vasodilatory, decapeptide angiotensin I to the vasoconstrictor angiotensin II within the RAAS (Figure 1). ACE-I also catalyzes the degradation of the vasodilatory compound bradykinin, which results in increased blood pressure [18]. ACE-I inhibitory peptides have been isolated from numerous sources including dairy products such as fermented yoghurts and cheese [19,20], marine co-product proteins [21], in particular collagen from fish skins [22], meat by-products [23], soy [24], hemp seed [25], Chinese and Iranian traditional medicines [26], vegetables including cruciferous vegetables such as broccoli [27], cereals [28] and micro and macroalgae [29,30]. ACE-I inhibitory peptides act on sub-sites of the active site of ACE-I via the C-terminal tri-peptide sequence at the end of a peptide. Many authors have highlighted the importance of the affinity of ACE-I competitive inhibitors to ACE-I of hydrophobic, aromatic or bulky branched side chain amino acid residues. The presence of C-terminal amino acids with a positive charge on the ε-amino group can also contribute to the potency of ACE-I inhibition [31]. Molecular weight is also an important attribute to consider when designing ACE-I inhibitory peptides. In general, ACE-I inhibitory peptides are short sequences of hydrophobic amino acids, and have low molecular weights. In order to determine if ACE-I inhibitory peptides are competitive or non-competitive, it is necessary to determine the minimum quantity of the peptide that inhibits the enzyme by 50% (the IC_50_ value of the peptide) and to assess the rate of inhibition using the Michaelis Menton equation and Lineweaver-Burk plots [32].

**Table 1:** Bioactive peptides derived from food sources and their use as bioactives in commercial products.

Peptide Sequence	Observed Bioactivity	Product	Producers of Product	Product Type	Co-Product Source	Reference
LKPNM	Antihypertensive	PeptACE™	Natural Factors Nutritional Products Ltd., British Columbia, Canada	Capsules	Bonito	[32]
LKPNM	Antihypertensive	Vasotensin^®^	Metagenics, USA	Tablet	Bonito	[32]
LKPNM	Antihypertensive	Levenorm^®^	Ocean Nutrition Canada Ltd., Nova Scotia, Canada	N/A	Bonito	[32]
LKPNM	Antihypertensive	Peptide ACE 3000	Nippon Supplement Inc., Osaka, Japan	Capsules	Bonito	[32]
LKPNM	Antihypertensive	Peptide Tea	Nippon Supplement Inc., Osaka, Japan	Powder	Bonito	[33]
VY	Antihypertensive	Lapis Support	Tokiwa Yakuhin Co., Ltd., Tokyo, Japan	Beverage	Sardine	[33]
VY	Antihypertensive	Valtyron^®^	Senmi Ekisu Co., Ltd., Ohzu-City, Japan	Ingredient	Sardine	[33]
FY, VY and IY	Antihypertensive	Wakame Jelly	Riken Vitamin, Tokyo, Japan	Jelly	*Undaria pinnatifida* (seaweed)	[34]
AKYSY	Antihypertensive	Peptide Nori S	Riken Vitamin, Tokyo, Japan	Beverage	*Porphyra yezoensis* (seaweed)	[35]
AKYSY	Antihypertensive	Mainichi Kaisai Nori	Shirako Co., Ltd., Numazu City, Japan	Powder	*Porphyra yezoensis* (seaweed)	[35]
IPP and VPP	Antihypertensive	Ameal S 120	Calpis Co., Ltd., Tokyo, Japan	Beverage	Milk	[36]
IPP and VPP	Antihypertensive	Ameal S	Calpis Co., Ltd., Tokyo, Japan	Tablet	Milk	[36]
IPP and VPP	Antihypertensive	Evolus^®^	Valio Ltd., Helsinki, Finland	Beverage	Milk	[36]
VY	Antihypertensive	Sato Marine Super P	Sato Pharmaceutical Co., Ltd., Tokyo, Japan	Tablet	Sardine	[33]
FFVAPFPEVFGK	Antihypertensive	Casein DP Peptio Drink	Kracie Pharmaceutical, Tokyo, Japan	Beverage	Milk	[37]
FFVAPFPEVFGK	Antihypertensive	C12 Peption	DMV International, Veghel, The Netherlands	Ingredient	Milk	[37]
LVY	Antihypertensive	Goma Pepucha	Suntory Beverage & Food Ltd., Tokyo, Japan	Beverage	Sesame	[38]
Numerous peptides	Antihypertensive	Bunaharitake	Yakult Health Foods Co., Ltd., Tokyo, Japan	Powder	Mushroom	[39]
VY, IY, IVY	Antihypertensive	StayBalance RJ	Api Co., Ltd., Gifu-City, Japan	Beverage	Royal jelly	[40]
VVYP	Weight management	Seishou-sabou	Moringa & Co., Ltd., Kanagawa, Japan	Beverage	Blood (bovine and porcine)	none
CSPHP	Cholesterol-lowering	Remake CholesterolBlock	Kyowa Hakko, Tokyo, Japan	Beverage	Soy	[41]
YLGYLEQLLR	Stress-relief	Lactium^®^	Ingredia, Arras Cedex, France	Beverage and capsules	Milk	[42]
N/A	Stress-relief	Stabilium^®^ 200	Yalacta, Caen, France	Capsules	Fish	[43]
N/A	Stress-relief	AntiStress 24	Forte Pharma Laboratories, France	Capsules	Fish	[43]
N/A	Stress-relief	Protizen^®^	Copalis Sea Solutions, Boulogne-sur-mer, France	Powder	Fish	[43]
N/A	Joint health	CH-Alpha^®^	Gelita Health Products GmbH, Eberbach, Germany	Beverage	Bovine collagen	
N/A	Joint health	Peptan^®^	Rousselot SAS, Angoulême, France	Powder	Bovine collagen	[44]
N/A	Joint health	Collagen HM	Copalis Sea Solutions, Portel France	Powder	Fish collagen	[45]
N/A	Joint health	Glycollagen^®^	Copalis Sea Solutions, Portel, France	Powder	Skate collagen	[45]
N/A	Immunomodulatory	PeptiBal™	InnoVactiv Inc., Rimouski, QC, Canada	Capsules	Shark	[46]
N/A	Gastrointestinal health	Seacure^®^	Proper Nutrition, USA	Capsules	Fish	[47]
N/A	Obesity and mental health	Douchi – traditional Chinese soybean product	Traditional Chinese medicine product, Hong Kong, China	N/A	N/A	[48]
N/A	Chinese sufu (fermented tofu)	Traditional product	Traditional Chinese medicine product, Hong Kong, China	N/A	N/A	[49]
Whey peptides	Blood pressure regulation and cholesterol control	BioZate^®^3 hydrolysed whey protein	Davisco Foods, Minnesota, MN, USA	Powder product	Whey proteins	[50]
Whey peptides	Blood pressure regulation	BioZate (1) hydrolysed whey protein	Davisco Foods, Minnesota, MN, USA	Powder product	Whey proteins	[51]
Fish collagen peptides	Skin health	Deyan, China	Deyan, Hubei, China	Powder product	Fish scale collagen peptides	[52]
Carnosine and Anserine	Antioxidant and anti-aging	Nivea Q-10 cream, Nivea	Nivea, France	Cream product	Meat muscle protein (beef and chicken)	[53]

#### 2.1.2. Sources and Structure of Renin Inhibitory Peptides

The enzyme renin (also known as angiotensinogenase) was first reported by Tigerstedt and Bergman [52] in 1898 when they observed that an extract from rabbit kidney increased blood pressure in rabbits. Renin is a member of the aspartic protease family, which also includes the enzymes pepsin, cathepsin, and chymosin. It is a monospecific enzyme that displays specificity for its only known substrate, angiotensinogen [53]. It is found primarily in the granular cells of the juxtaglomerular apparatus situated in the macula densa mechanism of the kidneys and is produced in response to three main stimuli: (i) Decrease in arterial blood pressure; (ii) Decrease in sodium chloride (NaCl) levels in the ultrafiltrate of the nephron in the kidneys; and (iii) sympathetic nervous system activities which also control blood pressure levels. Renin is produced through the activation of Pro-renin, the enzymatic precursor of Renin. Pro-renin is inactive due to a 43 amino acid N-terminal pro-peptide that covers the active site and blocks access of the active site to angiotensinogen. It is activated either through proteolytic cleavage of the pro-peptide chain or by non-proteolytic activation in the juxtaglomerular cells by the unfolding of the proteolytic pro-peptide, which is how the majority of circulating renin is produced. Renin inhibition is the rate limiting step within the RAAS. However, compared to ACE-I inhibitors, few renin inhibitory peptides have been discovered from food or natural products. Peptides generated following enzymatic hydrolysis of flaxseed fractions were found to inhibit both human recombinant renin and ACE [54]. Li and Aluko identified the renin inhibitory peptide with the amino acid sequence IR from fractions of pea protein isolates with IC_50_ values <25 mM [55]. More recently, Fitzgerald and colleagues identified the renin inhibitory tridecapeptide IRLIIVLMPILMA from the red seaweed *Palmaria palmata* [56]. Renin inhibitory peptides were also isolated from buckwheat protein, cereals and rice [57] and hemp seed previously.

In contrast to ACE-I inhibitors, renin inhibitors cause an increase in renin release (Figure 1). This is because they interrupt the negative feedback-mediated regulation of renin release. The combination of an ACE-I inhibitor, angiotensin receptor antagonist or renin inhibitor with another drug from these groups, or with a diuretic, markedly amplifies the increase in renin concentrations. Increases in the concentration of renin concentrations may be 100-fold, which then offsets the inhibition of the renin-angiotensin system by these antihypertensive drugs [58,59]. This may attenuate any reduction in blood pressure.

#### 2.1.3. Sources of Platelet Activating Factor Acetylhydrolase (PAF-AH) Inhibitory Peptides

Platelet activating factor acetylhydrolase (PAF-AH) plays a role in atherosclerosis and inflammation and this is thought to be due to the formation of lysophosphatidyl choline and oxidized non-esterified fatty acids. This enzyme is considered a promising therapeutic target for the prevention of atherosclerosis [60]. Recently, our group isolated the PAF-AH inhibitory tetrapeptide NIGK from the red seaweed *Palmaria palmata*. The dried powdered alga was hydrolyzed using the food grade enzyme papain, and the resultant peptide containing fraction generated using RP-HPLC. The peptide NIGK had an IC_50_ value of 2.32 mM [61].

#### 2.1.4. Sources of Dipeptidyl Dipeptidase IV (DPP-IV) Inhibitory Peptides

Inhibition of the enzyme dipeptidyl peptidase-IV (DPP-IV; EC 3.4.14.5) has potential in the prevention of diseases related to the development of metabolic syndrome including type-2 diabetes, heart health and obesity. DPP-IV degrades and inactivates glucagon-like peptide-1 (GLP-1) and gastric-inhibitory peptide (GIP), two incretin hormones which contribute to the enhancement of glucose-induced insulin secretion [62]. Recently, gelatin hydrolysis of fish skins were suggested as a good source of natural inhibitors of dipeptidyl peptidase-IV and could potentially be used in the management of type 2-diabetes and/or neuropathological disorders [63]. GLP-1 is released from intestinal L-cells in response to food entering the GI tract [62]. Collated data suggests that specific macronutrient constituents found in dairy foods act as secretagogues for GLP-1. Tryptophan is considered an important amino acid in the sequence of peptides with potential to inhibit DPP-IV [63]. In a recent study, twenty seven tryptophan containing dipeptides were evaluated for their ability to inhibit dipeptidyl peptidase IV (DPP-IV). Novel DPP-IV inhibitors were identified comprising of three potent dipeptides (Trp-Arg, Trp-Lys and Trp-Leu) with half maximum inhibitory concentration (IC_50_ values) <45 μM. With the exception of Leu-Trp which was approximately 20 times less potent than Trp-Leu, their reverse peptide did not inhibit DPP-IV. Trp-Asp was the only peptide studied herein with an *N*-terminal Trp residue which was not a DPP-IV inhibitor [64].

## 3. Peptides in Mental Health and Prevention of Diabetes PEP/POP Enzyme

### 3.1. Sources and Mechanisms of Action of Prolyl oligopeptidase (POP) or Prolyl endopeptidase (PEP) (EC 3.4.21.26) Inhibitory Peptides

POP or PEP is a proline-specific endopeptidase that is expressed in the brain and is known to cleave neuroactive peptides implicated in memory, learning and also in neurodegeneration. It is highly conserved and cleaves peptide bonds at the carboxyl side of Proline residues in proteins with a relatively small molecular weight (30 amino acids in size) containing the recognition sequence X-Pro-Y, where X is a peptide or protected amino acid and Y is either an amide, a peptide, an amino acid, an aromatic amine or an alcohol [65]. Furthermore, it is thought that POP may be involved in thalamocortical neurotransmission, memory and learning functions of hippocampal formation and GABAergic regulation of voluntary and in the development of Multiple Sclerosis (MS). Welches and colleagues found that POP is also a major component of the enzymatic pathways that participate in angiotensin metabolism in canine hypothalamus. Several POP inhibitors have been isolated from microbes, medical plants and foods or have been chemically synthesised in recent times and their anti-amnesic effects have been studied in rat models. Sørensen and colleagues [66] found that peptide fractions generated from cod, salmon and trout hydrolysates, autolysates, and water-soluble extracts of cheeses inhibited POP in hydrolysing Z-Gly-Pro-amidomethylcoumarin [66]. Natural POP inhibitors have also been isolated from wine [67], caseins [68], unsaturated fatty acid [69] and plant phenolics [70]. More recently, a protease treated sample of “Barquillo” was found to inhibit POP *in vitro*. “Barquillo” is a by-product obtained from cocoa processing by pressing and rolling cocoa butter. It is of high biological value due to its high protein content of between 20%–27% [71].

### 3.2. Sources of DPP-IV Inhibitory Peptides

Diets rich in specific bio-functional ingredients, including food protein derived peptides, have emerged as a potential strategy for the prevention and management of type-2-diabetes (T2D). It is thought that the number of persons diagnosed with diabetes will increase from 285 to 439 million by 2030 [3]. Blood vessel damage in the brain of patients with diabetes and high cholesterol can lead to symptoms of Alzheimer’s disease (AD) and prevention of diabetes and high cholesterol can help to reduce the risk of developing AD [72]. Glucagon-like peptide 1 (GLP-1) and glucose dependent insulinotropic polypeptide (GIP) both control blood glucose levels in the body. These are degraded by DPP-IV and several research groups are looking at the development of DPP-IV inhibitory agents to control glucose and prevent T2D [72,73,74,75]. Pharmaceutical DPP-IV inhibitory drugs available today include Saxagliptin (Onglyza™), and vildagliptin (Galvus^®^). These drugs do have some side effects including urinary and upper tract infections. Food derived bioactive peptides that inhibit DPP-IV provide an alternative for the potential prevention and treatment of both T2D and AD. Recently, Amylin, a pancreatic peptide, 37 amino acids in length which passes through the blood brain barrier easily provided the template for the amylin analog pramlintide which serves as an effective drug in the clinical treatment of T2D [72]. Furthermore, when injected, this peptide reduced behavioural impairment and brain amyloid pathology in murine models of Alzheimer’s disease.

Peptides derived from dairy, salmon [74], tuna [76], rice [77], amaranth and lysozyme proteins were found previously to inhibit DPP-IV *in vitro*. Kannan identified a pentapeptide from a bran rice hydrolysate which showed enhanced anti-Alzheimer’s activity. Few *in vivo* studies with DPP-IV inhibitors have been carried out to date in relation to their possible role in the prevention of AD. Several recent reports however have identified that dipeptidyl DPP-IV inhibitors have suppressive effects on atherosclerosis in apolipoprotein E-null (*Apoe*^−/−^) mice. Furthermore, the protective effects of the DPP-IV inhibitor sitagliptin in the blood-retinal barrier in a T2D animal model were shown previously [77].

### 3.3. Acetylcholinesterase Inhibitory Peptides (AChE Inhibitory Peptides)

The enzyme AChE (EC 3.1.1.7), present in the Central Nervous System (CNS) catalyzes the hydrolysis of Acetylcholine (Ach) to choline. ACh is released in the synaptic cleft, where it activates both postsynaptic and presynaptic cholinergic receptors, which results in cognition improvement [78,79]. AChE—a cholinesterase enzyme-terminates this neural-stimulating activity Protein hydrolysates are also a source of AchE inhibitory peptides. Tuna liver is a fish by-product and is normally discarded and/or used as fish and animal feed due to poor functionality. In a study carried out by Ahn and colleagues, tuna fractionated hydrolysates produced by the commercial enzymes alcalase, neutrase and protamex following flavourzyme hydrolysis showed excellent antioxidant activities against DPPH [76]. Furthermore, all fractionated hydrolysates inhibited acetylcholinesterase activity and the high MW fractions showed greater AChE inhibitory activities than LMW fractions [76]. The AChE inhibitory activity of Douchi—a traditional Chinese salt-fermented soybean food was examined and observed inhibition was attributed bioactive peptides generated from soybean protein following fermentation [80]. Similarly, the AChE inhibitory activity of Chinese sufu (fermented tofu) was observed by Chen and colleagues [81]. In a further study which examined the anti-obesity and anti-Alzheimer’s effect of rice bran, bioactive peptides <5 kDa in size were identified [71].

## 4. Bioactive Carbohydrates

Bioactive carbohydrates include prebiotics and immune cell regulators. Sources include polysaccharides from marine and terrestrial plants and animals and include fish mucus coating, Phaeophyceae and dairy products. Marine macroalgae or seaweeds are a rich source of commercially available polysaccharides which are used as functional ingredients in food manufacture [82]. Seaweeds, are classified according to their pigmentation into brown (Phaeophyceae), red (Rhodophyta), and green (Chlorophyta). The species of seaweeds that are industrially used belong to the divisions Rhodophyta and Phaeophyta. Bioactivities associated with seaweed polysaccharides include anti-thrombotic, anti-viral, antimicrobial and antioxidant properties.

These bioactivities are thought to be due to sulphated polysaccharides, which are not found in terrestrial plants and which may have specific functions in ionic regulation. Seaweed dietary fibers are particularly rich in soluble fractions, which in red seaweeds are mostly composed of sulphated galactans, such as agar or carrageenans. Soluble dietary fiber polysaccharides derived from brown seaweeds include the alginates, fucans, and laminaran [83]. These polymeric carbohydrates, or polysaccharides are non-toxic, have unique properties, such as their ability to form gels at low concentrations and have technofunctional applications [84].

### 4.1. Prebiotics

Prebiotics are non-digestible food ingredients that selectively stimulate the growth and/or activity of one or a limited number of beneficial bacteria (probiotics) in the colon [85]. Prebiotic carbohydrates currently available include fructooligosaccharides, lactulose, inulin and galactooligosaccharides from lactose (GOS-La) [86]. Currently, there is considerable interest in the discovery of new carbohydrates with potential prebiotic properties including galactooligosaccharides from lactulose (GOS-Lu). In order to be defined as prebiotic carbohydrates the carbohydrate must fulfill certain criteria [67,87]. The carbohydrate must firstly be resistant to gastric acidity, hydrolysis by mammalian enzymes, gastrointestinal absorption and fermentation by intestinal microflora [87]; Secondly, the carbohydrate in question must have selective stimulation on the growth and/or activity of intestinal probiotic bacteria [87]. As is the case with all functional foods, the final demonstration of a prebiotic effect must be carried out *in vivo* using the appropriate species. Recently O’Sullivan and colleagues examined the potential of seaweed polysaccharides as prebiotics [10]. Potential prebiotic carbohydrates from plant sources include soybean oligosaccharides, glucooligosaccharides, cyclodextrins, gentiooligosaccharide, germinated barley, oligodextrans, glucuronic acid, pectic oligosaccharide and whole grains [88].

#### 4.1.1. Prebiotics Carbohydrates in Brown Seaweeds

Sulfated fucans derived from brown seaweeds such as *Ascophyllum nodosum* and *Fucus* species are frequently referred to as fucans, fucosan, fucansulphate or fucoidans and were first isolated by Killing in 1913 [89]. These polysaccharides consist mainly of sulphated l-fucose and are easily extracted from the cell wall of brown algae with hot water or acid solutions. Fucans can make up greater than 40% of the dry weight of isolated cell walls. There are different types of sulphated fucans which include ascophyllan or xylofucoglycuronan. This polysaccharide is based on a backbone of uronic acid (mannuronic acid) with fucose containing branches (3-*O*-d-xylosyl-l-fucose-4-sulfate). Sargassans or glycuronofucoglycan are also sulphated fucans, based on linear chains of d-galactose with branches of l-fucose-3-sulfate or occasionally uronic acid. The International Union of Pure and Applied Chemistry (IUPAC) recommend defining sulphated fucan as a polysaccharide based mainly on sulphated l-fucose, with less than 10% of other monosaccharides [82]. The polysaccharides known as alginates are used in the food industry as thickeners, stabilizers and gelling agents and in recent decades, alginates (E400–E404) have become widely adopted as dietetic food hydrocolloids. *Ascophyllum nodosum* is the brown seaweed that is most exploited for alginate content worldwide. Studies regarding the dietary effects of alginates on faecal microbial fauna in male volunteers have shown that daily administration of 10 g of Sodium alginate for 2 weeks significantly increases the content of Bifidobacteria, while the number of *Enterobacteriacea* and the frequency of the occurrence of lectinase-negative *Clostridia* tend to decrease [90].

#### 4.1.2. Prebiotic Carbohydrates in Green Seaweeds

In the green algae, most work has focused on polysaccharides known as ulvans as they display several biological activities of potential interest for therapeutic, nutraceutical and personal care applications. Ulvans are poorly or not degraded by faecal bacteria and therefore could serve as stabilizers and promoters for the binding of growth factors to the high affinity receptors of the cells in the intestinal membrane [91]. A polysaccharide from the green marine algae *Ulva lactuca* was isolated and was studied for antiviral activity *in vitro* against a number of human and avian influenza viruses and considerable inhibition detected [92].

#### 4.1.3. Prebiotic Carbohydrates in Red Seaweeds

Sulphated galactans are found in the thallus of red seaweeds. Carrageenans are sulphated galactans extracted from seaweeds of the order Gigartinales, and from some genera of Dumontiales, Halymeniales and Rhodymeniales [82]. Agarans are sulphated galactans in which the α-galactose or 3,6-anhydro-α-galactose units belong to the l-series. Agarose which is biosynthesized by members of the orders Gelidiales and Gracilariales is an agaran polysaccharide. Many reports exist regarding the anticoagulant activity of carrageenan [82]. The species *Chondrus crispus* is the primary source of λ-carrageenan, whereas *Eucheuma cottoni* and *E. spinosum* are the sources of κ- and ι-carrageenans, respectively. The principal basis of the anticoagulant activity of carrageenan appeared to be an anti-thrombotic property [83]. λ-carrageenan showed greater anti-thrombotic activity than κ-carrageenan probably due to its higher sulphate content, whereas the activity of the un-fractionated material remained between the two. Λ-carrageenan consistently prolonged the clotting time and was more toxic than κ-carrageenan [84]. The toxicity of carrageenan depends on the molecular weight. The effect of carrageenan on human platelets was also examined previously. *Ex vivo*, carrageenan had an anticoagulant effect and inhibited platelet aggregation. The mechanism of anticoagulant activity of λ-carrageenan is exhibited via thrombin inhibition [84].

## 5. Bioactive Carbohydrates from Plant and Food Processing Co-Products

### 5.1. Chitin

Chitin is a naturally occurring biopolymer and derivatives include chitosan and chitooligosaccharides as well as glucosamine [8,9]. It is the major structural component of mushroom stalks (in particular Basidomycete mushrooms) and shell waste such as crab and prawn [8,9]. Chitin is a high molecular weight, linear polymer of *N*-acetyl-d-glucosamine units and can be easily processed into many other bioactive derivatives. Chitosan (CTS) results from the removal of considerable amounts of acetyl groups from chitin. CTS is water soluble and is a positively charged, heteropolymer of d-glucosamine. Both chitin and CTS are user friendly, non-toxic, biocompatible and biodegradable in nature. In the United States CTS was approved by the FDA for certain food applications including as an edible film to protect foods [85]. CTS produced by the Norwegian company Primex^®^ has Generally Recognized as Safe (GRAS) approval and is recognized as a functional food while chitosan-glucan isolated from *Aspergillus niger* obtained a novel food health claim from EFSA in 2011 [86]. CTS is known to lower density lipoprotein-cholesterol [87]. The positive charge nature of CTS and COS along with its polyelectrolyte properties, gel-forming abilities and the presence of reactive functional groups govern most of their biological activities. Other bioactivities associated with chitin and chitosan include ACE-I inhibition, antimicrobial and anti-obesity bioactivities [8,9].

### 5.2. Plant Sourced Prebiotic Oligoscaccharides, Obesity and Hypertension Prevention

The oligosaccharides known as raffinose which are isolated from the lupin seeds (*Lupinus albus* var. Multolupa) are known bifidogenic prebiotics and have been used during the manufacture of probiotic fermented milk [88]. Studies have shown that selection of *B. lactis* Bb-12 and *Lactobacillus acidophilus* in a mixed culture at a 1:1 ratio and addition of raffinose oligosaccharides to produce a fermented milk product would have the advantages of rapid growth and acidification rate and would likely increase the probiotic effect of the final functional product [88].

Soluble, plant derived prebiotics such as pectin, konjac mannan and modified starches are soluble in solutions and have viscous effects. These physicochemical properties have been found to affect physiological responses such as the lowering of blood cholesterol and increasing satiety due to delayed gastric emptying [89]. Fiber is associated with a reduction of obesity following consumption. Decreasing obesity by consumption of prebiotics could help to prevent the incidence of hypertension [90]. Various other mechanisms have been postulated to explain the ability of prebiotics to reduce the risk of high blood pressure. The lipid and cholesterol lowering effects of prebiotics could be attributed to the production of short chain fatty acids [91]. Prebiotics resist digestion in the small intestine and in the colon are fermented by microflora to produce short chain fatty acids (SCFAs). SCFAs produced in the bowel are absorbed in the portal vein and subsequently affect various metabolic processes. Propionate for example, could hinder fatty acid and cholesterol synthesis and lactate produced in the bowel cold play a role in lowering the synthesis of triaclyglycerol fatty acids [91].

## 6. Dairy Prebiotic Oligosaccharides

The dairy prebiotics market directly benefits from the growth in probiotics. Prebiotics from dairy sources include lactulose and galactooligosaccharides.

### 6.1. Lactulose

Lactulose is produced from lactose through alkali isomerization of the glucose moiety of lactose to fructose, thereby making it a combination of fructose and galactose and it cannot be degraded by mammals [92]. Lactulose is known to have prebiotic effects and may be used in that capacity or as a low-calorie sweetener. It is also used as a pharmaceutical agent to prevent constipation and in patients with hepatic encephalopathy to reduce blood ammonia concentration. Lactulose can selectively stimulate Bifidobacteria and Lactobacilli populations and is considered a prebiotic.

### 6.2. Galactooligosaccharides (GOS)

GOS is produced from lactose. It is heat and acid stable during storage unlike other prebiotics such as fructooligosaccharide (FOS). GOS are defined as a mixture of those substances produced from lactose, comprising between 2 and 8 saccharide units, with one of these units being a terminal glucose and the remaining saccharide units being galactose and disaccharides comprising 2 units of galactose. Three health claims on GOS have passed EFSA’s pre-screening, namely: “maintains a healthy normal digestive system”, “prebiotic/bifidogenic”, and “calcium absorption” [93].

## 7. Downstream Processing

Downstream processing of bioactive peptides and carbohydrates involves the recovery and purification of bioactive peptides and carbohydrates. Traditionally bioactive compounds have been extracted using methods which require use of solvent, thermal and/or physical methods. These methods are generally time and energy intensive. In addition, many bioactive compounds are sensitive to high temperature heat and the use of solvents. Recent research has focused on the development of novel extraction techniques that are more efficient in terms of yield, time, and costs and in addition are environmentally friendly and can better preserve the activity of target compounds. Extraction technologies which use ultrasound, microwaves, enzymes, supercritical fluid and pressurized liquid have been researched for extraction of bioactive compounds for food and pharmaceutical applications. Extraction of bioactive carbohydrates and peptides generally involves grinding, precipitation in an acid or basic medium followed by filtration and drying. Application of both classical and novel technologies for the extraction of bioactive compounds from various sources has been reviewed extensively [94,95]. Novel processing technologies has been employed for cell structure disintegration, thus facilitating the extraction of bioactive compounds from complex matrix whereas in some cases novel techniques when used in combination with enzymes enhance digestion reaction rates. Pre-treatment of samples using novel techniques followed by conventional extraction have been demonstrated to enhance extraction yields of bioactive carbohydrates and recovery of proteins for the production of bioactive peptides. Following section outlines application of novel technologies for enhancing reaction rates and cell disruption are discussed.

### 7.1. High Pressure Processing

High pressure processing (HPP) is a novel technique which employs pressure in the range of 100–800 MPa or even up to 1000 MPa. HPP is applied to enhance solvent permeability and facilitates extraction of bioactives from various matrices. Increase in cell permeability occurs due to the large differential pressure between cell internal and exterior of cell membranes. Increase in cell permeability allows solvent penetration, dissolution rates and improves mass transfer rates. Application of HPP followed by enzymatic hydrolysis or in the presence of enzyme has been investigated to enhance proteolytic hydrolysis of various protein sources. Hydrolysis of food proteins under HPP and enzymes including chymotrypsin, trypsin, and pepsin has been reported to enhance proteolytic reaction and changes the proteolytic pattern [96]. HPP is also reported to allow rapid removal of intact proteins leading to a reduction in binding properties as observed in the case β-lactoglobulins [97] and Ovalbumin [98]. The effect of HPP on whole egg white was examined using *in vitro* pepsin digestion and proteomic methods. Pepsin incubations conducted with an enzyme to protein ratio of 3:1 following high pressure treatment (400–800 MPa and 9 °C) resulted in increased hydrolysis of egg white proteins. HPP treatment of egg white at 800 MPa resulted in greater susceptibility to pepsin hydrolysis compared to thermal treatment at 95 °C. HPP treatment is also reported to improve antioxidant properties of enzymatic pea protein hydrolysates compared to heat treatment alone [99]. In a study, Garcia-Mora *et al*. [100] studied the effect of high pressure on hydrolytic efficiency of commercial protein enzymes and they observed that the proteolysis at 300 MPa led to a complete degradation of lentil proteins and increased peptides (<3 kDa). HPP causes protein conformational changes leading to a partial or full unfolding of polypeptides resulting in exposing peptides responsible for antioxidant activity. Unfolding of proteins and opening of protein structure also allows improved hydrolysis of proteins using proteolytic enyzymes.

### 7.2. Ultrasound Processing

Application of ultrasound (above 20 kHz) to enhance enzymatic reaction and/or extraction yield has been reported extensively. There are two main types of ultrasound equipment which can be employed, namely an ultrasonic water bath and an ultrasonic probe system fitted with horn transducers [100]. Ultrasonic water baths are relatively inexpensive and are commonly used to sonicate laboratory samples. Ultrasonic probe system with horn transducers introduces vibrations directly into the sample and may be used in batch or continuous mode [101]. The main driving force for the extraction effects of ultrasound is acoustic cavitation. Phenomenon of the creation, expansion, and implosive collapse of microbubbles in ultrasonically irradiated liquids is known as “acoustic cavitation”. The formation and collapse of cavitation bubbles generates macro-turbulence, high-velocity inter-particle collisions, and agitation in micro-porous particles of the biomass [102] allows disintegration of matrix. Impingement by these micro-jets results in surface peeling, erosion, and particle break down facilitating release of bioactive compounds from the biological matrix thus increasing extraction efficiency by improving mass transfer [103,104]. Ultrasound in combination with other conventional extraction techniques has been demonstrated as a potential technique for extraction of bioactive compounds [105]. Ultrasound can be carried out at low temperature, which facilitates the extraction of thermo-labile compounds with minimal damage, preserving bioactivity. Ultrasound can be employed with a wide range of solvents including aqueous extraction of bioactive compounds, *i.e*., for water-soluble components [106]. Application of ultrasound with enzymes has been demonstrated to improve extraction yields of bioactives by facilitating an increase in collisions between enzyme and substrate. Enhanced enzymatic activity of amylases, glucose oxidase, cellulases, dextranase has been reported [107,108]. Studies show that the low intensity ultrasound enhances reaction rates by enhancing both the catalytic and specificity constants, altering composition and modifications of α-helix and β-sheet fractions [107]. Application of ultrasound along with enzymes has a potential to enhance extraction yields of polysachararides from various plant matrices including wheat bran [108,109].

### 7.3. Microwave Assisted Extraction

Microwave assisted extraction involves the use of electromagnetic radiation in a frequency ranging from 300 MHz to 300 GHz to heat solvents in contact with a sample to separate compounds of interest from the sample matrix. Microwave possess both electric and magnetic field and allow heating of solvent and the sample via dipolar rotation and ionic conduction. The use of microwave energy in extraction process allows disruption of weak hydrogen due to dipole rotation of the molecules and improves solvent penetration into the matrix and thus facilitates the solvation [110]. Microwave assisted extraction has been reported to enhance the extraction yield of bioactives from various matrices compared to traditional solid liquid extraction [111]. The use of microwave assisted extraction may induce degradation of bioactive carbohydrates due to localised high temperature [112]. However, improved antioxidant capacity of bioactive polysaccharides from *Auricularia auricular* (AAP) using microwave assisted extraction has been reported [113]. Microwave assisted extraction has also been applied for extraction of bioactive sulfated polysaccharides from seaweed including *Fucus vesiculosus* [114] and *Ascophyllum nodosum* [115]. Microwave assisted acid hydrolysis of proteins rapid protein degradation for peptide mass mapping and tandem mass spectrometric analysis of peptides has been reported [116].

### 7.4. Supercritical Solvent Extraction

Efficiency of traditional solid liquid extraction process can be improved by applying pressure and/or temperature to improve extraction yields. Solvent properties including density, diffusivity and viscosity can be controlled with an application of pressure and temperature thus allowing the use of environmentally friendly solvents (e.g., water). Application of pressure and temperature can also improves penetration of solvent and assist in disruption of cellular matrix. For example, CO_2_ at a temperature and pressure above the critical point (31.06 °C and 7.38 MPa) becomes supercritical fluid. Supercritical CO_2_ has low viscosity which improves diffusivity and extraction yields [117,118,119]. Supercritical CO_2_ can be used for the extraction of polar and neutral compounds which can eliminate the use of organic solvents. Extraction efficiency of water can be improved by employing high temperature and pressure. Use of pressure in the range of 3.5 to 20 MPa and temperature in the range of 50–200 °C for liquids is also known as pressurised liquid extraction or accelerated solvent extraction, high pressure solvent extraction, pressurized fluid extraction, and enhanced solvent extraction (ESE) [120,121,122]. The temperature and pressure conditions employed in PLE are in the range of 50 to 200 °C and 3.5 to 20 MPa respectively. Application of pressure with solvents for the extraction of bioactive carbohydrates has been reported extensively [123,124].

## 8. Conclusions

Bioactive peptides and carbohydrates have huge potential for use as functional foods and exist already in several food and pharmaceutical products as techno-functional and bioactive ingredients. There are requirements for chemical and biotechnological methods concerning efficient, sustainable and economic drying, extraction and bio-refinery procedures for protein/peptide and carbohydrate isolation, hydrolysate generation, purification, up-scaling of production and compound identification methods. Downstream processing for high valued products such as peptides currently involves one or several energy-expensive procedures including drying, cellular disruption and extraction of bioactive molecules. Quality protein/peptide and co-products production/cost relation studies with different drying, extraction and purification methods are needed. Biomass pre-treatment methods including sonic-treatments and centrifugation need to be fully explored to ensure processing costs remain viable and maximum yields of bioactive compounds are obtained.

Peptides, proteins and carbohydrates can contribute to increase the production of healthy and sustainably produced food. At present, novel processing technology utilization is limited by a number of factors including: (1) affordable technologies for the optimized processing of protein/peptide-rich biomass; (2) lack of sustainability and high costs associated with pre-treatment and processing and extraction of proteins; (3) costs associated with the isolation and downstream processing of valuable protein/peptide isolates, hydrolysates and co-products; (4) of health and safety legislation. The bioactivities of existing bioactive peptides and carbohydrates should be examined in different assay systems as often known peptides with specific bioactivities can have multifunctional attributes. In addition, the toxicity and absorption, distribution, metabolism and excretion (ADME) profiles of known peptides with health beneficial activities should be examined.

Application of novel extraction technologies alone or in combination with other conventional techniques demonstrates the possibility of enhancing extraction yield of bioactives while preserving biological activities. However, the scale up to industrial applications still needs to be explored and optimized for large scale production of bioactives.
